# Do attachment-related differences in reflective functioning explain associations between expressed emotion and youth self-harm?

**DOI:** 10.1007/s12144-022-03614-w

**Published:** 2022-08-26

**Authors:** Jamie Kennedy-Turner, Vilas Sawrikar, Lucy Clark, Helen Griffiths

**Affiliations:** 1 Clinical Psychology Department, School of Health in Social Science, The University of Edinburgh, Doorway 6, Elsie Inglis Quad, Old Medical School, Teviot Place, Edinburgh, EH8 9AG UK; 2 Pennywell All Care Centre, CAMHS North Edinburgh, 1 Macmillan Crescent, Edinburgh, EH4 4WL UK; 3 NHS Education for Scotland, 102 West Port, Edinburgh, EH3 9DN UK

**Keywords:** Youth, Self-harm, Reflective functioning, Expressed emotion, Attachment

## Abstract

Youth self-harm is associated with poor health outcomes and attempted and completed suicide. Associations exist between self-harm and expressed emotion (EE), attachment insecurity, and reflective functioning (RF), but these associations are poorly understood. This study evaluates a mediation model in which perceived caregiver EE (pEE) exerts an indirect effect on youth self-harm through attachment insecurity and RF uncertainty. 461 participants aged 16–24 years completed an online survey. Statistical analyses revealed significant direct effects of pEE on attachment insecurity, and of RF uncertainty on self-harm; however, some direct effects were specific to pEE from female caregivers, and attachment insecurity in youth relationships with female caregivers. A significant direct effect of pEE on self-harm was found for pEE from male caregivers only. Significant indirect effects of pEE on self-harm through attachment anxiety and RF uncertainty were found only in relation to female caregivers. The findings encourage family-, attachment-, and mentalization-based approaches to preventing and treating youth self-harm, with a recommendation that caregivers are given adequate support, education, and skills-based training following youth disclosures of self-harm.

**Supplementary Information:**

The online version contains supplementary material available at 10.1007/s12144-022-03614-w.

## Introduction

Self-harm can be defined as “any behaviour resulting in self-poisoning or self-injury carried out by an individual, irrespective of motivation or the extent of suicidal intent (excluding accidents, substance misuse and eating disorders)” (p.6, Teuton et al., 2014). Self-harm is a major public health concern. Research evidence highlights significant associations between self-harm and attempted and completed suicide (Andover et al., 2012), hospital admissions (Hawton et al., 2007) and increased healthcare costs (Sinclair et al., 2011). Self-harm is also associated with other negative outcomes, including increased all-cause mortality (Morgan et al., 2017); violent and non-violent crime, and alcohol and substance use (Ohlis et al., 2020); low self-esteem, anxiety, and depression (Hawton, 2002); and eating disorders (Koutek et al., 2016). Self-harm is especially common among young people, with average onset between 12–14 years of age and lifetime prevalence rates estimated at 13.8–16% and higher rates reported in females (Cipriano et al., 2017). Better understanding of the development of youth self-harm is therefore warranted, to inform public health strategies and identify targets for clinical intervention.

Previous research demonstrates that some of young people’s experiences in family relationships, such as perceived maltreatment and parent–child relational trauma, are associated with increased risk of self-harm (Martin et al., 2016). Parental communication style is also relevant, with harsh punishment, invalidation, and conflict all associated with deficits in emotion regulation skills and increased self-harm in adolescence (Adrian et al., 2018; Sim et al., 2009). EE is a key measure of family emotional climate which assesses a caregiver’s negative attitudes, behaviours, and communication regarding family members (Brown et al., 1972). Narrative measures of EE (e.g. Camberwell Family Interview [CFI], Five Minute Speech Sample [FMSS]) involve trained observers coding a transcript given by a parent or caregiver about a target family member for specific patterns of communication; for example, criticism or hostility. However, researchers have noted conceptual issues with solely relying on narrative measures in examining risk for psychopathology among younger populations (Peris & Miklowitz, 2015). For instance, narrative EE measures were developed in the context of adult mental health. It is therefore unclear whether high EE represents atypical or maladaptive family patterns for children and adolescents, or whether high EE in families of young people is normative. Use of narrative measures alone may therefore not be developmentally suitable to assess the impact of family emotional climate on youth self-harm.

Furthermore, youth’s own experiences of EE from caregivers may be misrepresented or overlooked using traditional narrative measures, as these methods rely on coding EE from a transcript given by a family member, rather than any direct assessment or interview with the target individual. A growing body of research has examined youth’s perceived EE (pEE; Hale et al., 2007; Nelis et al., 2011), which provides insight into youth’s internal, subjective experiences of EE from their caregiver(s) using self-report measures such as the Level of Expressed Emotion scale (LEE; Cole & Kazarian, 1988). Several studies demonstrate that self-reported pEE has significant positive associations with youth self-harm (see Yates et al., 2008; Hack & Martin, 2018), emphasising that pEE is important to understanding familial environment-related risk in youth self-harm. Better understanding of the association between pEE and self-harm is identified as an important research direction, to support the development of evidence-based psychological treatments for youth self-harm, where family environment represents a salient risk factor (Peris & Miklowitz, 2015).

Attachment theory with consideration of attachment-related differences in reflective functioning (RF) offers a developmental framework for understanding the psychological processes linking pEE and self-harm. Attachment theory posits that early relational experiences with caregivers are internalised as internal working models (IWMs) of attachment-related stimuli and behaviour, which influence how individuals behave in subsequent relationships (Bowlby, 1969). Attachment experiences and resulting internalised attachment-related schema are thought to be central to the development of emotion regulation skills (Brumariu, 2015), with those with secure attachment representations having a greater capacity to regulate emotion using adaptive coping techniques, such as self-soothing or seeking social support. Conversely, those with insecure attachment representations have a reduced capacity to self-regulate and greater reliance on maladaptive coping strategies, such as self-harm, substance use, or social withdrawal (Kimball & Diddams, 2007).

As discussed above, attachment representations have a lasting influence on how individuals regulate emotion and relate to others. Theory and research into mentalization also indicates that individuals with insecure attachment representations are also more likely to have deficits in RF (Badoud et al., 2018). RF is a facet of mentalization and refers to the capacity to understand behaviour in oneself and others as motivated by internal experiences, such as thoughts and feelings, and is theorised to develop in the context of early attachment relationships (Fonagy et al., 1998). Typically, impairments in RF are characterised hypermentalizing (or excessive certainty) or hypomentalizing (or excessive uncertainty) about the internal states of the self and others, with genuine RF thought to lie between these two positions (Fonagy et al., 2016). Insecure attachment schema and related differences in RF may provide an explanation linking pEE and self-harm in young people, as both attachment security and RF are implicated in affect self-regulation (Jurist & Meehan, 2008; Kivity et al., 2021; Mikulincer & Shaver, 2012), with RF dysfunction, in particular RF uncertainty (RFu) or hypomentalization, associated with self-harm (Badoud et al., 2015; Cucchi et al., 2018; Fonagy et al., 2016). It is currently unclear as to why RFu appears to be particularly associated with self-harm, though Cucchi et al. (2018) suggest that a lack of adaptive certainty about internal states may contribute in some way to this increased risk. RF impairments are frequently reported in individuals diagnosed with borderline personality disorder (Katznelson, 2014), for which mentalization-based therapy (MBT) has an emerging evidence base (Vogt & Norman, 2019) and self-harm is a core diagnostic criterion (American Psychiatric Association, 2013). Furthermore, research indicates that parental RF and child outcomes are linked, with well-developed parental RF associated with improved mentalizing abilities in children (Camoirano, 2017) and greater attachment security (Fonagy et al., 1991; Fonagy et al., 1995; Katznelson, 2014), and impaired parental RF associated with emotion regulation impairments, externalising behaviours and anxiety disorders (Camoirano, 2017), outcomes with lasting impacts on young people’s development as they age.

**Fig. 1:**
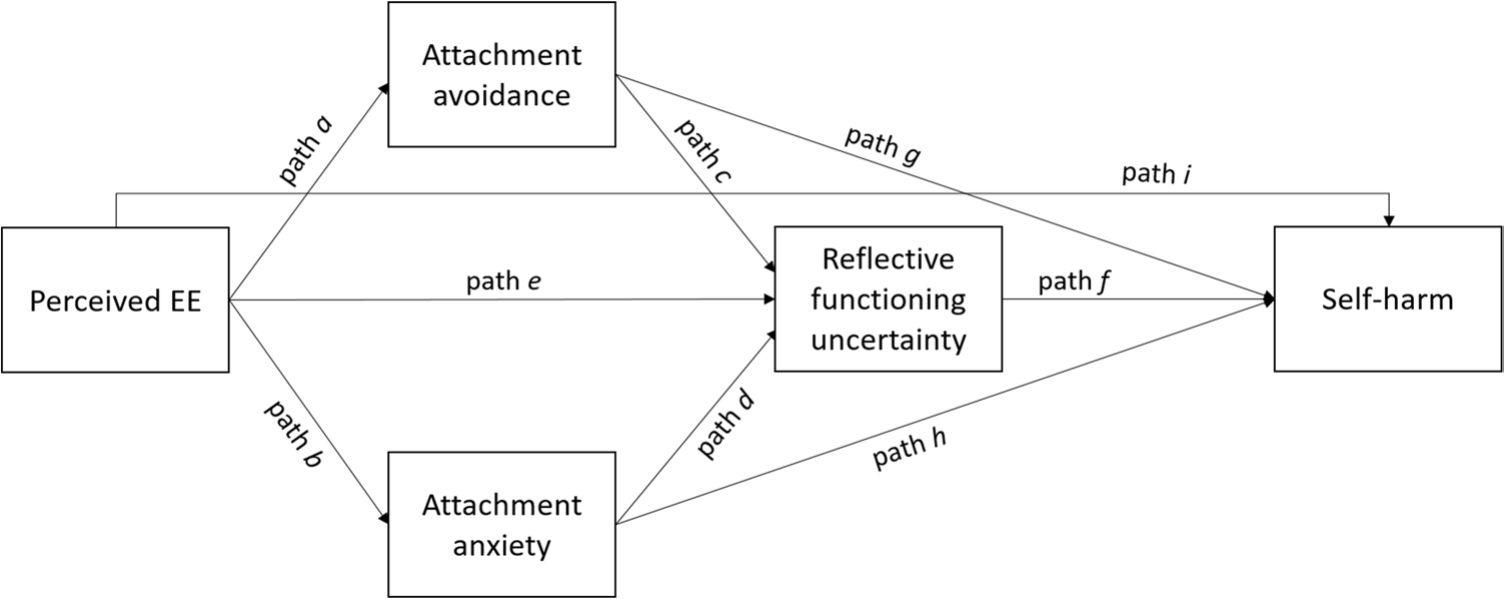
Diagrammatic representation of the theoretical model of youth self-harm

As discussed above, youth’s perception of familial expressed emotion (EE) is a known risk factor for youth self-harm (Michelson & Bhugra, 2012). However, limited research exists exploring why youth’s perception of familial EE is associated with increased self-harm risk. The current study used mediation analysis to assess the utility of examining attachment-related differences in RF to better understand how youth’s perceptions of familial EE are related to self-harm. The role of insecure attachment schema and RF is argued to be particularly important to understanding the role of pEE in youth self-harm, as it may give information about the youth’s internal working model of their family environment. To that end, the aim of the current paper was to evaluate a mediation model with consideration given to attachment insecurity and RF (see Fig. 1). No previous study has examined the potential for attachment-related differences in RF to mediate associations between pEE and youth self-harm. However, previous research findings provide preliminary support for individual pathways that constitute mediation effects. For instance, positive associations are reported between parental EE and attachment insecurity in young people, providing support for paths *a* and *b* (see Green et al., 2007; Jacobsen et al., 2000). Higher levels of parental EE are also associated with self-harm in childhood, adolescence and adulthood (see Allison et al., 1995; Hack & Martin, 2018; James & Gibb, 2019; Santos et al., 2009; Shields et al., 1994; Wedig & Nock, 2007; Yates et al., 2008), providing support for path *i*. Interestingly, most of these studies focused on maternal EE, therefore further research is needed to determine if these associations are specific to female caregivers or consistent across young people’s attachments to male caregivers as well, given the literature on gender-specific differences in young people’s attachment to mother and father (Diener et al., 2008). Attachment insecurity is also associated with increased lifetime prevalence and repetition of self-harm, providing support for paths *g* and *h* (Falgares et al., 2017; Rogier et al., 2017). However, it is unclear if self-harm is associated more strongly with a specific dimension of insecurity (i.e. avoidance or anxiety; Gormley & McNiel, 2010; Tatnell et al., 2014). Associations between attachment security and RF (paths *c* and *d*) have also been established in previous research (Badoud et al., 2018; see Katznelson, 2014, for a review), as have associations between RF and self-harm (path *f*; Badoud et al., 2015; Fonagy et al., 2016; Cucchi et al., 2018). The current study extends these findings by testing the mediating effect of insecure attachment schema and related differences in RF on the relationship between pEE and self-harm among young people.

### Hypotheses

This study hypothesises that pEE will be associated with youth self-harm via mediating pathways involving attachment security and RFu. Two mediation hypotheses will be tested: 1) the effect of pEE on self-harm will be mediated by attachment insecurity; and 2) the effect of pEE on self-harm will be mediated by both attachment insecurity and RF. pEE, attachment insecurity, and RF are also modelled to exert direct effects on self-harm, consistent with previous research findings. The model considers separate mediating pathways via attachment anxiety and avoidance to investigate whether one dimension is more strongly associated with RF or self-harm. We also evaluate the model in relation to female and male caregivers separately, considering possible differences in EE and attachment representations with caregivers of different genders.

## Methods

### Study design

The current study was of cross-sectional survey design using internet mediated research (IMR) methodology. IMR procedures were implemented in accordance with the British Psychological Society guidelines (British Psychological Society, 2017) and broader literature on IMR in mental health research (Pitman et al., 2015). The survey included a content warning, withdrawal button on each page, and contact details of support services and websites for participants. Qualtrics XM (Provo, UT, https://www.qualtrics.com) was used to create, distribute, and collate responses to the survey. Data were collected following ethical guidelines provided by the University of Edinburgh School of Health in Social Science Research Ethics Committee. The University of Edinburgh School of Health in Social Science Research Ethics and Integrity Committee provided institutional ethical approval for this study to take place.

### Participants

Youth aged 16 to 24 years were eligible to participate. A total of 760 respondents clicked the survey link. Of these, 661 completed the electronic consent form. Participant responses were excluded if they omitted, declined or withdrew consent (*n* = 101), were ineligible to participate due to age (*n* = 4) or were missing a high level of data in their responses (*n* = 194) (i.e. participants who did not provide full data for any key variables listed below), leaving 461 responses. Table 1 summarises participant characteristics. Respondents had a mean age of 18.05 years and most identified as female (83.9%). The majority of participants were living at home with both identified caregivers (39.7%) or their first identified caregiver (32.5%). Caregivers were typically biological parents (95.2% and 83.8% for first and second identified caregivers respectively). Generally, participants identified a female as their first caregiver (92.6%). Of those who provided data for two caregivers, most identified their second caregiver as male (88.3%).

**Table 1:** Sample characteristics

Sample characteristics	Descriptive statistics
Age in years, mean (SD, range)	18.05 (± 2.42, 16–24)
Gender identity, *n/N* (%)	
Female	387 / 461 (83.9%)
Male	23 / 461 (5.0%)
Gender diverse identity	42 / 461 (9.1%)
Not reported	9 / 461 (2.0%)
Usual residence *n/N* (%)	
Family home with both carers	183 / 461 (39.7%)
Family home with first carer	150 / 461 (32.5%)
Family home with second carer	15 / 461 (3.3%)
Rented accommodation	48 / 461 (10.4%)
Student accommodation	30 / 461 (6.5%)
Own purchased property	12 / 461 (2.6%)
No fixed accommodation	5 / 461 (1.1%)
Other	18 /461 (3.9%)
No. of caregivers in survey, *n/N* (%)	
One	121 / 461 (26.2%)
Two	340 / 461 (73.8%)
PHQ-9, mean (SD, range, *n*)	17.75 (± 6.56, 0–27, *n* = 461)
GAD-7, mean (SD, range, *n*)	14.08 (± 5.50, 0–21, *n* = 460)

Abbreviations: PHQ− 9 – Patient Health Questionnaire; GAD−7 – Generalised Anxiety Disorder

### Procedures

The survey link was distributed via promotion and advertising on social media. Relevant charities and organisations were approached with requests to promote the link through their networks, social media pages, forums and websites. Snowball sampling was made possible from the debrief page, whereby respondents could copy and share the survey link should they wish. It was not possible to calculate response rates as complete data on the total numbers who viewed the various advertisements were not recorded. Once participants provided digital consent, they were directed to the online questionnaire battery. Participants chose to provide data regarding one or two of their primary caregivers, defined as ‘the person or people whom they felt were their most important caregiving figure(s), or the adult person or people with whom they spent the most time growing up’.

### Measures

#### Demographics questionnaire

Participants were asked their age, gender identity (e.g. male, female, transgender, non-binary, agender, genderqueer, prefer not to say, prefer to self-describe), and the number of caregivers for which they wished to provide data. Gender was asked as a multi-response question in an attempt to make the demographics questionnaire more inclusive of the diverse gender identities likely to be present in a sample of young people of this generation (Parker & Igielnik, 2020; Ruberg & Ruelos, 2020). Participants also provided information regarding caregiver gender identity and relationship (e.g., biological parent, adoptive or foster parent, guardian, etc.), and whether they were currently living with their identified caregiver(s). Caregiver-respondent relationship was dichotomised for correlational and regression analyses: “biological parent(1)/non-biological caregiver(0)”, and living with caregiver “yes(1)/no(0)”.

#### Perceived expressed emotion

pEE was assessed using the Level of Expressed Emotion scale (Cole & Kazarian, 1988; Hale et al., 2007). The LEE is a 38-item self-report measure which assesses pEE in target relatives from the respondent’s perspective. The LEE shows good internal consistency and convergent validity in adolescent populations (Hale et al., 2007; Nelis et al., 2011). Respondents rated how strongly they agree with statements about their caregiver(s) (e.g. ‘…gets annoyed when I want something from them’) on a scale of 1 to 4, with a total sum score from 38 to 152. Higher scores indicate higher levels of pEE. Participants completed the LEE for each identified caregiver. Internal consistencies for the LEE were high in this study (α_female_ = 0.96, α_male_ = 0.96).

#### Attachment insecurity

Attachment insecurity was assessed using the Experiences in Close Relationships – Relationship Structures questionnaire (ECR-RS; Fraley et al., 2011). ECR-RS is a 9-item self-report measure designed to assess individuals’ attachment security on two dimensions; anxiety (’I often worry that this person does not really care for me’) and avoidance (’I don’t feel comfortable opening up to this person’). Respondents rate how strongly they agree with the items on a scale of 1 to 7. A total score is calculated for each subscale using mean scores across items after reverse coding (anxiety: items 1 to 4). Higher scores indicate higher levels of attachment anxiety or avoidance. Internal consistencies for all subscales were high to excellent (α = 0.84—0.92).

#### Reflective functioning

RF was assessed using the Reflective Functioning Questionnaire, Short Version (RFQ-8; Fonagy et al., 2016; Ha et al., 2013). The RFQ-8 is an 8-item self-report questionnaire designed to assess the capacity of participants to reflect on the internal mental states of self and others. Participants indicated their agreement with questionnaire items by rating them on a 7-point Likert scale, ‘Strongly Disagree’ to ‘Strongly Agree’. The original RFQ-8 scoring procedure generates two subscales; hypomentalizing, or uncertainty about mental states; and hypermentalizing, or certainty about mental states (Fonagy et al., 2016). However, due to issues raised in recent psychometric evaluations of the RFQ-8 (Müller et al., 2022; Spitzer et al., 2021), which critique the use of double-scoring the same items to derive supposedly uncorrelated item residuals, and found that the RFQ-8 inadequately assesses a maladaptive form of hypermentalizing, the present study used RFQ-8 to assess a single latent factor of RFu (or hypomentalizing; e.g., ‘People’s thoughts are a mystery to me’). In line with recommendations by Müller et al. (2022) and Spitzer et al. (2021), this unidimensional RFu scale was calculated by reverse scoring item 7 to fit overall scale polarity (‘I always know what I feel’) and taking a mean response across all items. Internal consistency for RFQ-8 used in this study was acceptable (α = 0.76).

#### Youth self-harm

Youth self-harm was assessed using the 18-item self-harm subscale of the Risk-Taking and Self-Harm Inventory for Adolescents (RTSHIA-SH; Vrouva et al., 2010). This measure demonstrates high internal consistency, test–retest reliability and sufficient validity in adolescent samples (Vrouva et al., 2010). Respondents were asked to rate how often they have engaged in different self-harm behaviours (e.g.,’Have you ever intentionally cut your skin?’) on a scale of 0 to 3 (‘Never’, ‘Once’, ‘More than once’, ‘Many times’). Possible scores range from 0 to 54. Higher scores indicate a greater lifetime frequency of self-harm behaviours. Internal consistency for RTSHIA-SH was high in this study (α = 0.92).

#### Depression

Depressive symptoms were assessed using the Patient Health Questionnaire (PHQ-9; Kroenke et al., 2001), a 9-item self-report measure developed as a screening tool for depressive symptoms in the general population. Respondents rate how often they have experienced nine symptoms over the past two weeks (e.g., ‘Feeling down, depressed or hopeless’), scoring on a scale of 0 (‘Not at all’), 1 (‘Several days’), 2 (‘More than half the days’), and 3 (’Every day’). PHQ-9 has a range of possible total scores, from 0 to 27. Higher scores indicate greater frequency and severity of depressive symptomatology. Internal consistency for PHQ-9 was high in this study (α = 0.88).

#### Anxiety

Anxiety symptoms were assessed using the Generalised Anxiety Disorder questionnaire (GAD-7; Spitzer et al., 2006), a 7-item self-report measure developed as a screening tool for generalised anxiety disorder symptoms. Respondents rate how often they have experienced seven common anxiety symptoms in the past two weeks (e.g., ‘Feeling nervous, anxious, or on edge’), scoring on a scale of 0 to 3 (described for PHQ-9 above). Possible scores range from 0 to 21, with higher scores indicating greater frequency and severity of generalised anxiety symptomatology. Internal consistency for GAD-7 was high in this study (α = 0.89).

### Power calculation

An a priori power calculation was computed to determine an appropriate sample size for which to aim. Current best practice recommendations for estimating sample size and power for mediation analyses suggest using Monte Carlo power analysis (Muthén & Muthén, 2002; Thoemmes et al., 2010) and to test indirect effects using bootstrapped confidence intervals (Zhang, 2014). Performing Monte Carlo power analyses can be difficult, however, as it requires all population parameters to be specified for the model of interest, and parameters may not be known (Schoemann et al., 2017). It is also computationally intensive and specific software skills are required. The free web-based application developed by Schoemann et al. (2017) was therefore used to expedite this process. The web application was set up to estimate the sample size required for a mediation model with two serial mediators, with a conventional target power level of 0.8 and confidence intervals set at 95%, corresponding to α = 0.05. The range of possible sample sizes was set between 50 and 800. In line with recommendations by Mundform et al. (2011), the number of replications for the Monte Carlo power analysis was set to 5000, with 20,000 draws per replication (Schoemann et al., 2017). To generate data for the model in the web application, users must enter values that allow the application to compute a covariance matrix for all variables in the model. For the purposes of sample size calculation, correlation effect sizes of 0.3, considered medium by convention (Cohen, 1988), were assumed between all variables. Using the above assumptions, a minimum sample of n = 276 was recommended to achieve sufficient power to identify significant direct and indirect effects.

### Data analysis plan

Preliminary analyses were conducted to examine descriptive statistics and correlations between key study variables to determine which potential psychosocial demographic covariates (e.g. age, gender identity, depression, anxiety, whether participants were currently living with the caregiver, whether the caregiver was a biological parent) should be included in the path analysis. Descriptive statistics and variable correlations were computed using IBM SPSS Statistics for Windows (Version 27). Covariates were included in the model if correlations indicated that it was either a putative mental health or demographic risk factor for youth self-harm (e.g., *p*-value > 0.05). Two datasets were created for path analyses, evaluating mediation models for female and male caregivers separately; (i) female caregiver model (Model 1) and (ii) male caregiver model (Model 2). Missing data was evaluated using Little’s MCAR test in each dataset to determine patterns of missingness. Datasets for both Model 1 and 2 were tested to ensure that they satisfied the assumptions for hypothesis testing using linear regression, including linearity, normality of residuals, homoscedasticity of residuals, and independence of errors (Kenny, 2021). In both datasets, assumption testing indicated that the above assumptions were satisfactorily met (see Supplementary Information).

Serial mediation analyses with full information maximum likelihood (FIML) estimation were used to evaluate the proposed model using path analysis in IBM SPSS Amos (Version 27) (Arbuckle, 2020). In both models, pEE (total LEE score) was the exogenous variable and self-harm (total RTSHIA-SH score) was the outcome variable. Attachment anxiety (ECR-RS Anxiety) and attachment avoidance (ECR-RS Avoidance) were the first level of endogenous mediating variables. RFu (RFQ-8 total score) represented the second level of endogenous mediator. Each endogenous variable was regressed on the preceding variable. Covariate variables representing participant’s age, gender, and mental health (PHQ-9 and GAD-7 total score) were regressed onto all endogenous variables and were free to correlate with each other.

A multiple-index strategy was used to determine model fit, which included Chi-square goodness of fit, comparative fit index (CFI) and root mean square error of approximation (RMSEA) (Jackson et al., 2009). Consistent with recommendations for testing mediation (Yzerbyt et al., 2018), individual components of mediating pathways (‘direct effects’) were first tested for significance. Mediation was then assessed by the significance of indirect effects related to a-priori specified paths representing the main hypotheses of the study: (i) pEE → attachment insecurity (either attachment anxious or avoidant) → self-harm, or (ii) pEE → attachment insecurity (either attachment anxious or avoidant) → RFu → self-harm. Parameter estimates for direct and indirect effects were determined using bootstrapping with bias corrected confidence intervals with 10,000 resamples (Puth et al., 2015). Results were obtained using the Indirect Effects plugin for IBM SPSS Amos (Gaskin & Lim, 2018), which gives unstandardised and standardised regression estimates, p-values, and bootstrapped upper and lower confidence intervals. Significance testing was evaluated at α = 0.05.

## Results

### Descriptive statistics

Table 2 summarises descriptive characteristics for outcome measures used in the study. Correlation coefficients for variables in the female and male caregiver datasets are presented in Tables 7 and 8 in the Supplementary Information. Shapiro–Wilk tests revealed that all continuous data in both female and male caregiver datasets were non-normally distributed, with the exception of self-harm in the male caregiver dataset (*W*(221) = 0.989, *p* = 0.07), indicating that Spearman’s rho correlations would be appropriate. There were significant positive associations between identified predictor variables (pEE, attachment anxiety, attachment avoidance, RFu) and self-harm in both male and female caregiver datasets. Predictor variables all significantly correlated with each other. The strongest positive association between predictor variables was between LEE total score and attachment avoidance in both datasets.

**Table 2:** Descriptive statistics for main study variables

Variable	*n*	Mean (*SD*)	Min	Max
RFQ-8	461	5.02 (1.02)	2.13	7.00
RTSHIA-SH	461	25.45 (11.13)	0.00	51.00
ECR-RS				
Global attachment avoidance	460	5.01 (1.24)	1.33	7.00
Global attachment anxiety	460	5.90 (1.34)	1.00	7.00
Female caregiver attachment avoidance	361	4.62 (1.61)	1.00	7.00
Female caregiver attachment anxiety	361	2.98 (1.91)	1.00	7.00
Male caregiver attachment avoidance	221	5.10 (1.64)	1.00	7.00
Male caregiver attachment anxiety	221	3.32 (2.10)	1.00	7.00
LEE				
Female caregiver	361	91.38 (26.52)	44.00	146.00
Male caregiver	221	89.66 (26.43)	44.00	146.00

Abbreviations: RFQ−8 – Reflective Functioning Questionnaire, Short Version; RTSHIA−SH – Risk−Taking and Self−Harm Inventory for Adolescents – Self−Harm subscale; ECR−RS – Experience in Close Relationships – Relationship Structures; LEE – Level of Expressed Emotion scale

Regarding covariates, significant associations were found between both depression and anxiety, and pEE, attachment anxiety and avoidance, RFu, and self-harm in both datasets. Significant negative associations were observed between age and predictor and outcome variables, apart from attachment avoidance and LEE total score in the female caregiver dataset, and attachment anxiety in the male caregiver dataset, which were non-significant. Point-biserial correlations revealed no significant associations between self-harm and caregiver relationship as dichotomised into biological parent (1) vs. non-biological caregiver (0), and whether respondents were living with their caregiver (1) or not (0) in either data set (see Tables 9 and 10 in the Supplementary Information). Therefore, caregiver biological parent status and respondent-caregiver cohabitation status were not included as covariates for either model.

### Common method bias and multi-collinearity

Tests for common method bias (CMB) were initially conducted using Harman’s single factor test (Podsakoff et al, 2003). Harman’s test revealed that a single factor explained 40.10% of the variance in the female caregiver model and 42.41% in the male caregiver model. In both cases, this was below the conventional critical threshold (i.e., ≥ 50%) but above the more conservative critical threshold of 40% (Babin et al., 2016), therefore further tests were conducted to determine whether CMB was present. The correlation matrix indicated that no single correlation was found to be above 0.90, with the strongest association being 0.73 in the male caregiver dataset, between LEE and ECR MAAv (see Table 7 in the Supplementary Information), well below the critical threshold of 0.90 (Bagozzi et al., 1991). Further, a full multi-collinearity test was conducted for all constructs in both models. Variable inflation factors (VIF) in the female caregiver model ranged from 1.12 to 2.75; VIFs in the male caregiver model ranged from 1.12 to 2.75, all below the critical threshold of 3.3 (Kock & Lynn, 2012). This indicates that the collinearity between variables was not strong enough to warrant corrective measures and also suggests that CMB was not a confounding issue in either model.

### Missing data

Missing data were primarily associated with non-completion of a second caregiver dataset due to having only selected one caregiver about which to provide responses. In the datasets used for path analysis, missing data were missing completely at random for both the female caregiver dataset (Little’s MCAR test: χ^2^(1060, *n* = 361) = 24.30, p > 0.05) and male caregiver dataset (Little’s MCAR test: χ^2^(318, *n* = 221) = 76.50, p > 0.05), indicating that statistical imputation using FIML estimation would be appropriate (Schafer & Graham, 2002).

### Model fit statistics

Two serial mediation models were used for path analysis: (i) female caregiver model (Model 1) and (ii) male caregiver model (Model 2). Model 1 provided an excellent fit to the data (χ^2^(1) = 0.39, *p* = 0.53, CFI = 1.00, TLI = 1.02, RMSEA = 0.00, SRMR = 0.003), whereas Model 2 demonstrated a poor to moderate fit to the data (χ^2^(1) = 7.16, *p* = 0.01, CFI = 0.99, TLI = 0.69, RMSEA = 0.167, SRMR = 0.01) and should therefore be interpreted with caution. R-squared statistics showed that Model 1 explained 46.6% of the variance in self-harm, 30.2% of the variance in RFu, 47.0% of the variance in female caregiver attachment avoidance, and 28.4% of the variance in female caregiver attachment anxiety. Model 2 explained 48.8% of the variance in self-harm, 31.5% of the variance in RFu, 52.0% of the variance in male caregiver attachment avoidance, and 40.3% in male caregiver attachment anxiety.

### Model 1 – Youth reporting on female caregivers.

**Fig. 2:**
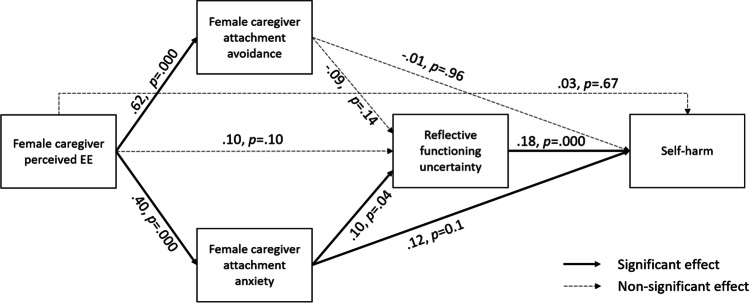
Path diagram of mediation analysis using female caregiver dataset testing indirect effects of pEE on youth self-harm via mediators. Notes: path coefficients represent standardised regression estimates; covariates and residuals have been omitted from diagram for ease of interpretation; covariates were age, PHQ-9 total score, GAD-7 total score, and participant gender (male, female, gender-diverse identity)

Figure 2 and Table 3 summarise the results of significance testing for direct effects in Model 1. pEE was significantly and positively associated with greater attachment avoidance (path *a*: β = 0.61; *p* = 0.000) and attachment anxiety (path *b*: β = 0.41; *p* = 0.000.) but was not significantly associated with RFu (path *e*: β = 0.10; *p* = 0.10) or self-harm (path *i*: β = 0.03; *p* = 0.67). Attachment anxiety was significantly and positively associated with RFu (path *d*: β = 0.10; *p* = 0.04), whereas attachment avoidance was not significantly associated with RFu (path *c*: β = -0.09; *p* = 0.14). Significant direct effects on self-harm were found from RFu (path *f*: β = 0.18; *p* = 0.000) and attachment anxiety (path *h*: β = 0.12; *p* = 0.01), but not attachment avoidance (path *g*: β = -0.01; *p* = 0.96). The pattern of direct effects indicated potential mediating pathways of 1) pEE → attachment anxiety → self-harm, and 2) pEE → attachment anxiety → RFu → self-harm (see Supplementary Information for a full description of direct effects in the female caregiver model). Table 3 shows the results for testing indirect effects in model 1. Results from testing indirect effects indicate that the effect of pEE on self-harm is accounted for via indirect effects through attachment anxiety (path *b* x *h*: β = 0.02; *p* = 0.01) and attachment anxiety and RFu (path *b* x *d* x *f*: β = 0.003; *p* = 0.03). Separate significant indirect effects were also obtained for pEE on RFu through attachment anxiety (path *b* x *d*: β = 0.002; *p* = 0.04) and for attachment anxiety on self-harm through RFu (path *d* x *f*: β = 0.11; *p* = 0.03). Indirect pathways through attachment avoidance were non-significant.

**Table 3:** Indirect effects in the serial mediation model using female caregiver dataset

Indirect path	Path labels	Indirect effect (SE)	Lower 2.5%	Upper 2.5%	p-value
LEE → ECR-RS-FAAv → RFQ-8	*a* x *c*	-.002 (.00)	-.01	.00	.13
LEE → ECR-RS-FAAv → RFQ-8 → RTSHIA-SH	*a* x *c* x *f*	-.004 (.00)	-.01	.00	.10
LEE → ECR-RS-FAAv → RTSHIA-SH	*a* x g	-.001 (.02)	-.03	.03	.96
LEE → ECR-RS-FAAnx → RFQ-8	*b* x *d*	.002 (.00)	.00	.00	.04*
LEE → ECR-RS-FAAnx → RFQ-8 → RTSHIA-SH	*b* x *d* x *f*	.003 (.00)	.00	.01	.03*
LEE → ECR-RS-FAAnx → RTSHIA-SH	*b* x *h*	.02 (.01)	.01	.04	.01**
LEE → RFQ-8 → RTSHIA-SH	*e* x *f*	.01 (.01)	-.00	.02	.07^┼^
ECR-RS-FAAv → RFQ-8 → RTSHIA-SH	*c* x *f*	-.011 (.08)	-.31	.02	.10^┼^
ECR-RS-FAAnx → RFQ-8 → RTSHIA-SH	*d* x *f*	.11 (.06)	.01	.25	.03*

Abbreviations: RFQ−8 – Reflective Functioning Questionnaire, Short Version; PHQ−9 – Patient Health Questionnaire; GAD−7 – Generalised Anxiety Disorder; RTSHIA−SH – Risk−Taking and Self−Harm Inventory for Adolescents – Self−Harm subscale; ECR−RS – Experience in Close Relationships – Relationship Structures; FAAv – Female caregiver attachment avoidance; FAAnx – Female caregiver attachment anxiety

┼*p* value <.10, * *p* value <.05, **p value <.01

### Model 2 – Youth reporting on male caregivers.

**Fig. 3:**
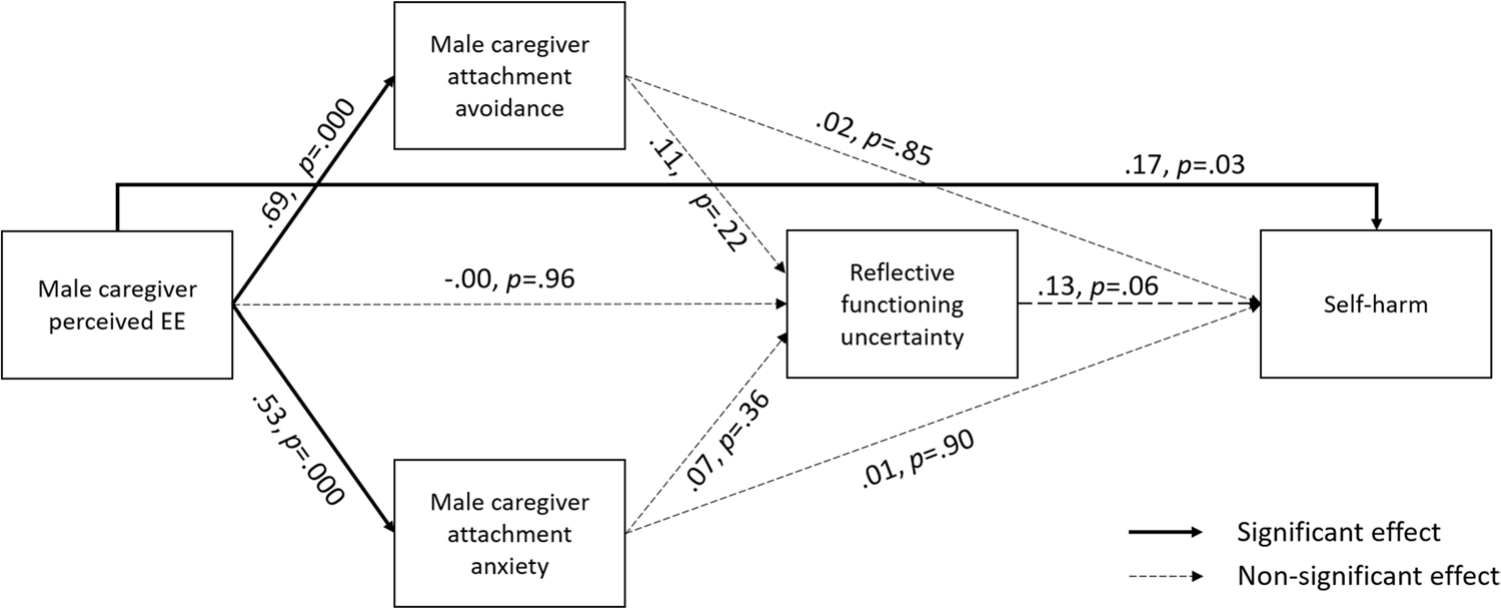
Path diagram of mediation analysis using male caregiver dataset testing indirect effects of perceived EE on youth self-harm via mediators. Notes: path coefficients represent standardised regression estimates; covariates and residuals have been omitted from diagram for ease of interpretation; covariates were age, PHQ-9 total score, GAD-7 total score, and participant gender (male, female, gender-diverse identity)

Figure 3 and Table 4 summarise the pattern of direct effects obtained for the male caregiver model. pEE significantly predicted attachment avoidance (path *a*: β = 0.69; *p* = 0.000), attachment anxiety (path *b*: β = 0.53; *p* = 0.000) and self-harm (path *i*: β = 0.17; *p* = 0.03) but did not significantly predict RFu (path *e*: β = .-00; *p* = 0.96). The direct effect of RFu on self-harm approached significance (path *f*: β = 0.11; *p* = 0.06), however, neither dimension of attachment insecurity significantly predicted RFu (path *c*: β = 0.11; *p* = 0.22; path *d*: β = 0.07; *p* = 0.36) or self-harm (path *g*: β = 0.02; *p* = 0.85; path *h*: β = 0.01; *p* = 0.90). Taken together, this indicates that, in contrast to the female caregiver model (Model 1), none of the hypothesised mediator variables exerted a significant direct effect on self-harm in the male caregiver model (Model 2; see the Supplementary Information for a full description of direct effects in the male caregiver model). As indicated on Table 4, no significant indirect effects were found in this model.

**Table 4:** Indirect effects in the serial mediation model using male caregiver dataset

Indirect path	Path labels	Indirect effect (SE)	Lower 2.5%	Upper 2.5%	p-value
LEE → ECR-RS-MAAv → RFQ-8	*a* x *c*	.00 (.00)	-.00	.01	.21
LEE → ECR-RS-MAAv → RFQ-8 → RTSHIA-SH	*a* x *c* x *f*	.00 (.00)	-.00	.02	.11
LEE → ECR-RS-MAAv → RTSHIA-SH	*a* x g	.00 (.02)	-.04	.05	.85
LEE → ECR-RS-MAAnx → RFQ-8	*b* x *d*	.00 (.00)	-.00	.00	.35
LEE → ECR-RS-MAAnx → RFQ-8 → RTSHIA-SH	*b* x *d* x *f*	.00 (.00)	-.00	.01	.23
LEE → ECR-RS-MAAnx → RTSHIA-SH	*b* x *h*	.00 (.02)	-.03	.03	.90
LEE → RFQ-8 → RTSHIA-SH	*e* x *f*	.00 (.01)	-.01	.01	.93
ECR-RS-MAAv → RFQ-8 → RTSHIA-SH	*c* x *f*	.08 (.08)	-.20	.35	.12
ECR-RS-MAAnx → RFQ-8 → RTSHIA-SH	*d* x *f*	.04 (.06)	-.03	.21	.23

Abbreviations: RFQ−8 – Reflective Functioning Questionnaire, Short Version; PHQ−9 – Patient Health Questionnaire; GAD−7 – Generalised Anxiety Disorder; RTSHIA−SH – Risk−Taking and Self−Harm Inventory for Adolescents – Self−Harm subscale; ECR−RS – Experience in Close Relationships – Relationship Structures; MAAv – Male caregiver attachment avoidance; MAAnx – Male caregiver attachment anxiety; LEE – Level of Expressed Emotion scale

## Discussion

This study evaluated a mediation model of self-harm, developed by synthesising research evidence of associations between self-harm and EE, attachment insecurity and RF. The model hypothesised that attachment representations and related differences in RF (i.e. greater RFu) mediated the association between pEE and self-harm among youth. The model was evaluated in relation to youth reporting on their relationships with female and male caregivers separately. Mediation hypotheses were only supported where youth reported on their relationship with a significant female caregiver; pEE exerted significant indirect effects on self-harm i) via attachment anxiety, and ii) via attachment anxiety and RFu. These indirect effects were significant after controlling for age and respondent gender, depression and anxiety. The results did not support the mediation hypotheses when youth reported on their relationship with a significant male caregiver.

This study provides further evidence of associations between pEE and self-harm in young people, as has been reported by a growing body of previous research (Allison et al., 1995; James & Gibb, 2019; Santos et al., 2009; Shields et al., 1994; Wedig & Nock, 2007). Various explanations for this association have been proposed. Michelson and Bhugra (2012) hypothesise that family environments high in EE are laden with negative affective experiences (e.g. criticism, hostility, intrusiveness, lack of emotional support), outside the adolescent’s control, leading to increased depression and hopelessness, and self-harm as a means of escape or communicating an interpersonal need (e.g., for rescue). This hypothesis fits with the Four Functions Model of non-suicidal self-injury, which hypothesises that self-harm is a self-reinforcing behaviour which serves intra- and inter-personal functions, such as regulating adverse affective experiences while signalling distress to, and eliciting caregiving from, others (Bentley et al., 2014). Speaking of evidenced associations between parental EE and youth psychopathology more broadly, Peris and Miklowitz (2015) hypothesise that EE could constitute a form of toxic family stress; when faced with symptoms of adolescent psychological disorder, caregivers who respond critically, intrusively, unsupportively or in a hostile manner may create a negative affective environment which serves to exacerbate symptoms of psychological distress.

Whatever the case may be, this study suggests that there may be differential effects of parental EE on self-harm in young people depending on the gender of the caregiver. Higher pEE from both caregivers increased self-reported youth self-harm, but appeared to affect young people via different processes. pEE from male caregivers exerted a direct effect on self-harm, whereas pEE from female caregivers exerted indirect effects through attachment anxiety, and attachment anxiety and RFu. These differential effects on the basis of parent gender are in keeping with broader literature indicating that there are gender-specific differences in mother–child and father-child attachment relationships (Diener et al., 2008), and that gender-specific effects mediate the relationship between mother/father attachment and self-injurious behaviour in young people (Tao et al., 2020). Given the low levels of male respondents in this study, further conclusions about the salience of same-gender versus opposite-gender parent–child dyads cannot be drawn from the data presented here.

This study also provides the first evidence that differences in attachment schema and RF explain why pEE is a risk factor for youth self-harm, at least when young people report on internal representations of attachment with a significant female caregiver. The results extend prior research findings implicating other mediators and moderators in the association between pEE and youth self-harm, with the roles of self-criticism (Wedig & Nock, 2007) and feelings of alienation from parents (Yates et al., 2008) having been previously identified. The finding of a significant mediating pathway involving an insecure anxious attachment representation and RFu is consistent with existing mentalization theory; those with insecure attachment representations are theorised to demonstrate greater fluctuations in their RF capacity throughout life, particularly when the attachment system is activated by interpersonal threats or stressors (e.g. negative communication from an attachment figure) (Fonagy & Luyten, 2009; Fonagy et al., 1998).

Importantly, the results suggest that maternal attachment insecurity, particularly attachment anxiety, is most influential in the developmental process by which attachment anxiety and RFu mediates the relationship between pEE and youth self-harm (Glazebrook et al., 2015). The results preliminarily suggest attachment to female caregivers is more salient for the development of RF and emotional regulation strategies than attachment to male caregivers. It may be the case that different processes mediate the association between pEE from male caregivers and youth self-harm. Again, this would be in line with the broader literature indicating that there are gender-specific attachment-related differences in mother–child and father-child attachment relationships (Diener et al., 2008), and more recent research indicating that different gender-specific effects mediate the relationship between mother/father attachment and self-injurious behaviour in young people (Tao et al., 2020). It is noted that failure to detect a significant indirect effect in the male caregiver model could alternatively be related to methodological factors. Analyses in the male caregiver dataset may have been underpowered due to sample size (*n* = 221). Of further note is that most female caregivers were identified as the first caregiver (92.3%) and most male caregivers identified as second caregiver (77.0%). This may indicate that most participants viewed the female caregiver as their primary attachment figure. It is therefore possible the significant indirect effects obtained were due to female caregivers representing the primary caregiver figure, rather than the effects being associated with caregiver gender alone. Thus, further research with larger samples reporting on male and female caregivers is required to determine whether there is a caregiver-gender specific effect in the association between pEE and youth self-harm.

Though the examination of direct effects in mediation analyses was not the primary focus of the current study, certain results warrant specific discussion in context of previous literature. pEE from both male and female caregivers exerted a significant direct effect on both dimensions of attachment insecurity, replicating previous research findings (Green et al., 2007; Jacobsen et al., 2000) and supporting existing theory that the nature and quality of family communication influences attachment security (Michelson & Bhugra, 2012), with communication perceived as irritable, intrusive, critical or lacking in emotional support (i.e. high EE) leading to reduced attachment security. However, there was no significant direct effect of attachment avoidance on youth self-harm in either caregiver model, replicating previous research findings that attachment anxiety is more strongly associated with self-harm than attachment avoidance (Gormley & McNiel, 2010; Tatnell et al., 2014). Attachment avoidance was also not associated with RFu in either model. While this is contrary to previous results reporting that both attachment anxiety and avoidance were significantly associated with RF certainty and uncertainty (Brugnera et al., 2021), this study relied on self-report from practicing psychotherapists which may not be comparable to self-report outcomes gathered directly from youth participants. Overall, the results emphasise the significance of inner representations of attachment anxiety as a process by which pEE increases risk for youth self-harm.

### Theoretical and clinical implications

Taken together, the above findings provide direct support to the growing body of research indicating that higher pEE has a deleterious effect on attachment security and youth self-harm, and is in line with broader findings indicating that observer-rated EE is associated with youth self-harm. The present study suggests that perceptions of caregiver communication as lacking in emotional support, intrusive, irritable and critical (pEE) is associated with increased frequency and severity of self-harm in young people, but the association between pEE and youth self-harm differs depending on the gender of the caregiver perceived as high in EE. Importantly, the increased risk for youth self-harm associated with pEE appears to be linked to developmental processes of insecure attachment schema with a significant female caregiver and related RFu, which have been implicated in maladaptive affect regulation via self-harm (Badoud et al., 2015; Fonagy et al., 2016; Katznelson, 2014; Mikulincer & Shaver, 2012). pEE may constitute a form of communication which is perceived as an attachment-salient interpersonal threat, causing an activation of anxious attachment representations, and a “switching off” of RF (Green et al., 2021), leading to greater RFu and leaving the individual vulnerable to affect dysregulation and self-harm as a means of coping (Fonagy & Bateman, 2015).

These findings emphasise the importance of taking a family-systems approach to working with young people presenting with self-harm. Family communication, specifically EE, may be targeted by clinical intervention (Garcia-Lopez et al., 2014), leading to improvements in attachment security and subsequent outcomes. Further, the finding that EE is associated with attachment security and indirectly with self-harm could inform public health strategies aimed at improving communication between caregivers and adolescents. Recent qualitative research indicates that commonly reported parental reactions to disclosures of self-harm diverge substantially from adolescent’s preferred or ideal responses (Curtis et al., 2018). The development of resources and training programmes for parents regarding supportive communication about self-harm, drawing on research into adolescent’s preferred reactions or ideal responses to disclosures of self-harm, could be beneficial for both parents and young people. For example, Curtis et al. (2018) highlight that attempts by parents to take control, increase monitoring and implement disciplinary practices are perceived by young people as unhelpful. Future work to develop parental resources and training programmes could include psychoeducation on self-harm, and information on adolescent’s preferred responses to self-harm disclosures. There is broad recognition in the literature that parents own mental health is impacted by child disclosures of self-harm, but that parents can be an invaluable source of support to young people and should be supported in helping young people to manage self-harm (Arbuthnott & Lewis, 2015).

Finally, this study provides preliminary support for the use of Mentalization-Based Therapy with young people who self-harm. A significant direct effect of RFu on self-harm was found in the female caregiver model and approached significance in the male caregiver model. It theoretically follows that reduced uncertainty about mental states may help adolescents reduce self-harm. A recent systematic review highlights MBT as a potentially effective therapeutic approach across a range of clinical presentations, including youth self-harm (Malda-Castillo et al., 2019). Our results tentatively suggest that involving parents, in particular female caregivers, in a supportive or co-therapist position in MBT work, may be beneficial. This could be through the adjunct of attachment-based interventions to MBT; psychoeducation for the female caregiver on self-harm to address any misconceptions or unhelpful attributions; and communication skills training for female caregivers aimed at reducing unhelpful communication patterns (e.g. patterns indicative of high EE). Though as noted above, RF capacity is theorised to reduce in the context of interpersonal threat or stress (Fonagy & Luyten, 2009; Fonagy et al., 1998), therefore involving a female caregiver high in EE to whom the adolescent is insecurely attached may present just such an interpersonal threat, and actually hinder the development of greater RF capacity. Future research on MBT effectiveness for treating youth self-harm could consider introducing one or multiple of the above caregiver components to treatment to determine whether these additions enhance therapeutic outcomes.

### Limitations

This study has several limitations. Firstly, the cross-sectional design precludes conclusions about causality. Future longitudinal studies could explore the longer-term developmental components of the reported associations. Secondly, there is a risk of bias due to the high proportion of respondents who were excluded due to missing data. Furthermore, the sample was self-selecting, and therefore likely to consist of individuals with an interest in the subject area or perhaps personal experience of the topic. An additional limitation is that most of the sample identified as female, and most caregiver data was provided regarding female biological parents. This limits the finding’s generalisability to other gender identities and attachment relationships. As mentioned above, research indicates gender-specific attachment differences between same-sex and different-sex parent–child dyads (e.g., Diener et al., 2008). Future studies should aim to recruit a more representative range of attachment relationships. The limited demographics collected lowered participant burden, but with the result that the lack of information about ethnicity, education, employment, and socioeconomic status makes the sample’s representativeness unknown. This study also focused on a late adolescent population and cannot draw any conclusions about earlier developmental stages. Further research should explore these associations in community samples of young people in the pre-, early or mid-adolescent period.

This study collected data using entirely self-report methods, increasing risk of bias due to CMB, though as reported above, statistical tests indicated that CMB was not present to a confounding degree in either model. Nonetheless, using validated observer-rated measures of EE, attachment and RF in adjunct to self-report measures may have enhanced the robustness of our findings. Further, it is important to acknowledge that model fit for the male caregiver model was poor. It may be that the fit indices were affected by the smaller sample size in the male caregiver model, though it is also possible that other variables mediate the relationship between pEE from male caregivers and youth self-harm, given the finding of a direct significant association obtained of male caregiver pEE on self-harm. Finally, national UK lockdowns were co-occurring with data collection due to the COVID-19 pandemic; this context may have resulted in a higher prevalence and severity of psychological symptoms in the general population (Newlove-Delgado et al., 2021), possibly reflected in this study by the high reported levels of depressive and anxious symptomatology.

### Conclusion

This study provides evidence for associations between youth self-harm and pEE, attachment anxiety and RF, and caregiver gender-specific effects within these associations. Specifically, the role of attachment anxiety and its association with RFu were identified as risk factors mediating the association between perceived EE from caregivers and youth self-harm. The obtained results highlight the potential role for family intervention and MBT in youth self-harm, as well as providing evidence to inform public health strategies around family communication. Further longitudinal research is implicated, to explore more fully the developmental aspects of the predictor variables associated with self-harm in late adolescence. The results obtained are encouraging, highlighting several constructs through which youth self-harm could be indirectly targeted and prevented or reduced.

## Supplementary Information

Below is the link to the electronic supplementary material.

Supplementary file1 (DOCX 1.20 MB)

## Data Availability

The datasets generated and analysed during the current study are not publicly available, as participants did not provide consent for their data to be made available on a public repository. The datasets are available from the corresponding author on reasonable request and subject to relevant ethical approvals.
