# Supplementary material for: Do attachment-related differences in reflective functioning explain associations between expressed emotion and youth self-harm?
Source: Curr Psychol. 2022 Aug 26:1–15. Online ahead of print. doi: 10.1007/s12144-022-03614-w (PMC9411045; doi:10.1007/s12144-022-03614-w)
Supplement: Supplementary file 1 — Supplementary file1 (DOCX 1.20 MB) [file 12144_2022_3614_MOESM1_ESM.docx]

Title: Do attachment-related differences in reflective functioning explain associations between expressed emotion and youth self-harm?

Journal name: Current Psychology

Authors (affiliations):
Dr Jamie Kennedy-Turner (The University of Edinburgh, NHS Lothian Child and Adolescent Mental Health Service)
Dr Vilas Sawrikar (The Universitry of Edinburgh)
Dr Lucy Clark (NHS Lothian Child and Adolescent Mental Health Service)
Dr Helen Griffiths (The University of Edinburgh, NHS Lothian Child and Adolescent Mental Health Service)

E-mail address of corresponding author: [Jamie.Kennedy-Turner@ed.ac.uk](mailto:Jamie.Kennedy-Turner@ed.ac.uk)

**Supplementary information**

**Direct effects.** Tables 5 and 6 present a full description of the direct effects obtained by testing the male and female caregiver mediation models.

Table 5. Direct effects in the serial mediation model using female caregiver dataset

| **Direct path** | **Path label** | **Estimate** | **Lower 2.5%** | **Upper 2.5%** | **p-value** |
| --- | --- | --- | --- | --- | --- |
| LEE → ECR-RS-FAAv | *a* | .61 | .54 | .68 | .000*** |
| LEE → ECR-RS-FAAnx | *b* | .41 | .31 | .49 | .000*** |
| ECR-RS-FAAv → RFQ-8 | *c* | -.09 | -.21 | .03 | .14 |
| ECR-RS-FAAnx → RFQ-8 | *d* | .10 | .00 | .20 | .04* |
| LEE → RFQ-8 | *e* | .10 | -.02 | .23 | .10^┼^ |
| RFQ-8 → RTSHIA-SH | *f* | .18 | .10 | .27 | .000*** |
| ECR-RS-FAAv → RTSHIA-SH | *g* | -.01 | -.12 | .11 | .96 |
| ECR-RS-FAAnx → RTSHIA-SH | *h* | .12 | .03 | .21 | .01** |
| LEE → RTSHIA-SH | *i* | .03 | -.10 | .15 | .67 |

^Abbreviations: RFQ-8 – Reflective Functioning Questionnaire, Short Version; PHQ-9 – Patient Health Questionnaire; GAD-7 – Generalised Anxiety Disorder; RTSHIA-SH – Risk-Taking and Self-Harm Inventory for Adolescents – Self-Harm subscale; ECR-RS – Experience in Close Relationships – Relationship Structures; FAAv – Female caregiver attachment avoidance; FAAnx – Female caregiver attachment anxiety; LEE – Level of Expressed Emotion scale.
┼^*^p^* ^value <.10, *^ *^p^* ^value <.05, **^*^p^* ^value <.01, ***^*^p^* ^value =.001^

Table 6. Direct effects in the serial mediation model using male caregiver dataset

| **Direct path** | **Path label** | **Estimate** | **Lower 2.5%** | **Upper 2.5%** | **p-value** |
| --- | --- | --- | --- | --- | --- |
| LEE → ECR-RS-MAAv | *a* | .69 | .61 | .76 | .000*** |
| LEE → ECR-RS-MAAnx | *b* | .53 | .42 | .63 | .000*** |
| ECR-RS-MAAv → RFQ-8 | *c* | .11 | -.06 | .27 | .22 |
| ECR-RS-MAAnx → RFQ-8 | *d* | .07 | -.08 | .21 | .36 |
| LEE → RFQ-8 | *e* | -.00 | -.19 | .18 | .96 |
| RFQ-8 → RTSHIA-SH | *f* | .11 | -.00 | .23 | .06^┼^ |
| ECR-RS-MAAv → RTSHIA-SH | *g* | .02 | -.13 | .16 | .85 |
| ECR-RS-MAAnx → RTSHIA-SH | *h* | .01 | -.12 | .14 | .90 |
| LEE → RTSHIA-SH | *i* | .17 | .02 | .33 | .03* |

^Abbreviations: RFQ-8 – Reflective Functioning Questionnaire, Short Version; PHQ-9 – Patient Health Questionnaire; GAD-7 – Generalised Anxiety Disorder; RTSHIA-SH – Risk-Taking and Self-Harm Inventory for Adolescents – Self-Harm subscale; ECR-RS – Experience in Close Relationships – Relationship Structures; MAAv – Male caregiver attachment avoidance; MAAnx – Male caregiver attachment anxiety; LEE – Level of Expressed Emotion scale.
┼^*^p^* ^value <.10, *^ *^p^* ^value <.05, ***^*^p^* ^value <.001^

**Correlation analyses.** Tables 7 to 10 present the correlation analyses completed.

|  | 1 | 2 | 3 | 4 | 5 | 6 | 7 | 8 |
| --- | --- | --- | --- | --- | --- | --- | --- | --- |
| 1. LEE | - |  |  |  |  |  |  |  |
| 1. ECR FAAv | .66** | - |  |  |  |  |  |  |
| 1. ECR FAAnx | .52** | .39** | - |  |  |  |  |  |
| 1. RFQ-8 | .24** | .15** | .27** | - |  |  |  |  |
| 1. RTSHIA-SH | .31** | .26** | .38** | .45** | - |  |  |  |
| 1. Age | -.08 | -.08 | -.20** | -.18** | -.15** | - |  |  |
| 1. PHQ-9 | .35** | .35** | .39** | .47** | .60** | -.26** | - |  |
| 1. GAD-7 | .24** | .21** | .31** | .47** | .48** | -.22** | .70** | - |
| Mean | 91.37 | 4.62 | 2.98 | 5.04 | 25.39 | 18.20 | 17.77 | 14.04 |
| SD | 26.52 | 1.61 | 1.91 | 1.00 | 11.04 | 2.48 | 6.65 | 5.59 |
| Minimum | 44.00 | 1.00 | 1.00 | 2.13 | .00 | 16.00 | .00 | .00 |
| Maximum | 146.00 | 7.00 | 7.00 | 7.00 | 51.00 | 24.00 | 27.00 | 21.00 |

Table 7. *Spearman’s rho correlations and descriptive statistics of variables and covariates in the female caregiver dataset*

^Abbreviations: LEE - Level of Expressed Emotion scale; ECR-RS - Experience in Close Relationships - Relationship Structures; FAAv - Female caregiver attachment avoidance; FAAnx - Female caregiver attachment anxiety; RFQ-8 - Reflective Functioning Questionnaire, Short Version; RTSHIA-SH - Risk-Taking and Self-Harm Inventory for Adolescents - Self-Harm subscale; PHQ-9 - Patient Health Questionnaire; GAD-7 - Generalised Anxiety Disorder.^

Table 8. *Spearman’s rho correlations and descriptive statistics of variables and covariates in the male caregiver dataset*

|  | 1 | 2 | 3 | 4 | 5 | 6 | 7 | 8 |
| --- | --- | --- | --- | --- | --- | --- | --- | --- |
| 1. LEE | - |  |  |  |  |  |  |  |
| 1. ECR MAAv | .73** | - |  |  |  |  |  |  |
| 1. ECR MAAnx | .61** | .55** | - |  |  |  |  |  |
| 1. RFQ-8 | .26** | .28** | .31** | - |  |  |  |  |
| 1. RTSHIA-SH | .37** | .31** | .39** | .44** | - |  |  |  |
| 1. Age | -.16* | -.17* | -.10 | -.24** | -.22** | - |  |  |
| 1. PHQ-9 | .28** | .27** | .37** | .48** | .63** | -.28** | - |  |
| 1. GAD-7 | .24** | .15* | .34** | .43** | .53** | -.27** | .67** | - |
| Mean | 89.66 | 5.10 | 3.32 | 5.02 | 24.10 | 18.40 | 17.22 | 13.85 |
| SD | 26.43 | 1.65 | 2.10 | .99 | 11.17 | 2.62 | 6.56 | 5.62 |
| Minimum | 44.00 | 1.00 | 1.00 | 2.25 | .00 | 16.00 | .00 | .00 |
| Maximum | 146.00 | 7.00 | 7.00 | 6.75 | 50.00 | 24.00 | 27.00 | 21.00 |

^Abbreviations: LEE - Level of Expressed Emotion scale; ECR-RS - Experience in Close Relationships - Relationship Structures; MAAv - Male caregiver attachment avoidance; MAAnx - Male caregiver attachment anxiety; RFQ-8 - Reflective Functioning Questionnaire, Short Version; RTSHIA-SH - Risk-Taking and Self-Harm Inventory for Adolescents - Self-Harm subscale; PHQ-9 - Patient Health Questionnaire; GAD-7 - Generalised Anxiety Disorder.^

Table 9. *Point-biserial correlations and descriptive statistics of dichotomous covariates and outcome variable in the female caregiver dataset*

|  | 1 | 2 | 3 |
| --- | --- | --- | --- |
| 1. RTSHIA-SH | - |  |  |
| 1. Biological parent | -.09 | - |  |
| 1. Living with carer | .10 | .06 | - |
| Mean | 25.39 | .95 | .72 |
| SD | 11.04 | .21 | .45 |
| Minimum | .00 | .00 | .00 |
| Maximum | 51.00 | 1.00 | 1.00 |

^Abbreviations: RTSHIA-SH - Risk-Taking and Self-Harm Inventory for Adolescents - Self-Harm subscale. N.B: variables 2 and 3 coded as follows: biological parent (1) vs. non-biological caregiver (0); respondents living with female caregiver (1) or not (0)^

Table 10. *Point-biserial correlations and descriptive statistics of dichotomous covariates and outcome variable in the male caregiver dataset*

|  | 1 | 2 | 3 |
| --- | --- | --- | --- |
| 1. RTSHIA-SH | - |  |  |
| 1. Biological parent | -.06 | - |  |
| 1. Living with carer | .01 | .03 | - |
| Mean | 24.10 | .91 | .59 |
| SD | 11.17 | .29 | .49 |
| Minimum | .00 | .00 | .00 |
| Maximum | 50.00 | 1.00 | 1.00 |

^Abbreviations: RTSHIA-SH - Risk-Taking and Self-Harm Inventory for Adolescents - Self-Harm subscale. N.B: variables 2 and 3 coded as follows: biological parent (1) vs. non-biological caregiver (0); respondents living with female caregiver (1) or not (0)^

**Assumptions testing**

**Linearity**

Figure 4. *Matrix scatter plot displaying linear relationships between predictor, mediator and outcome variables and covariates in the female caregiver dataset*


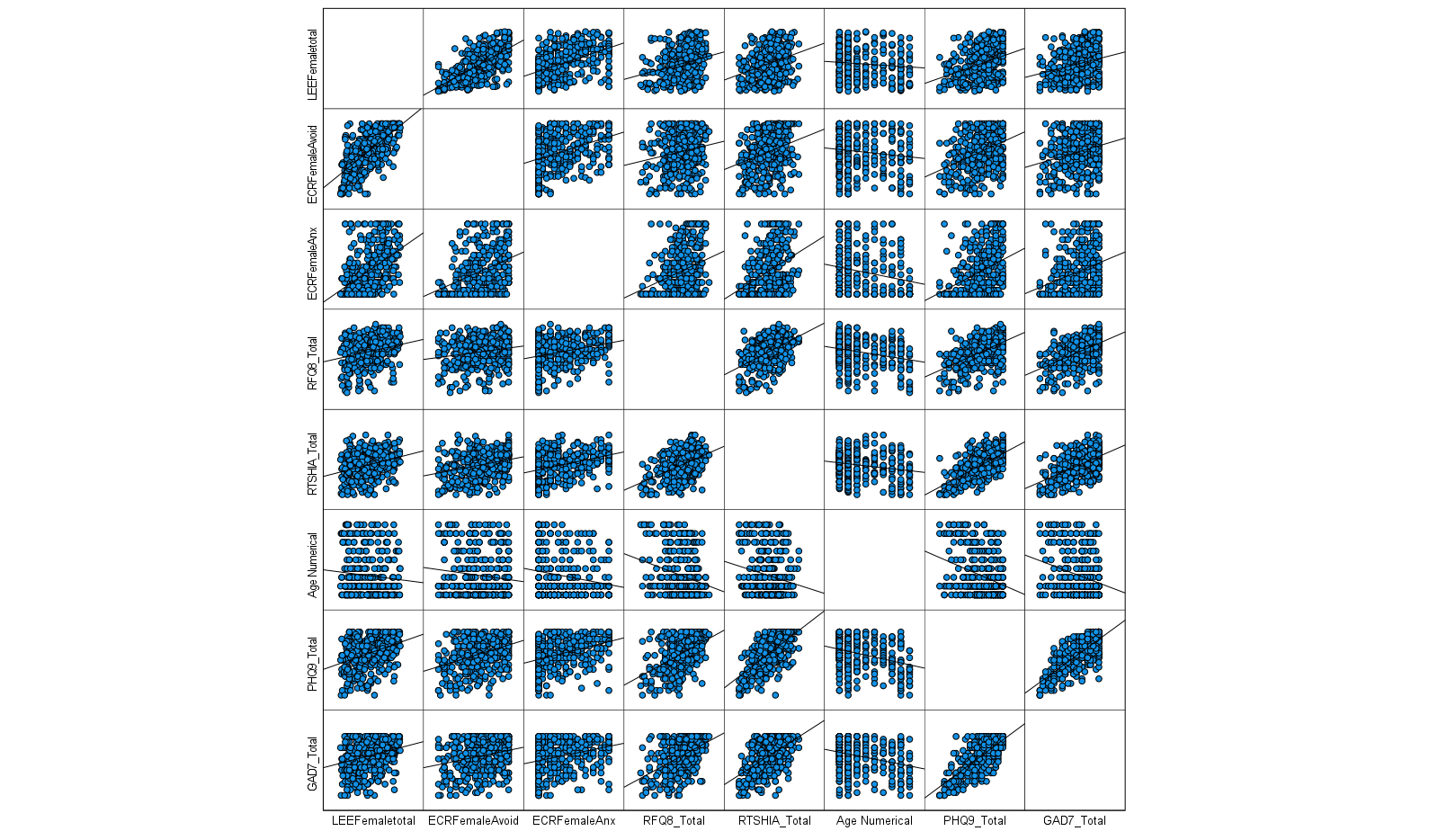


^N.B. Categorical covariate of gender not included in the scatterplot matrix.^

Figure 5. *Matrix scatter plot displaying linear relationships between predictor, mediator and outcome variables and covariates in the male caregiver dataset*


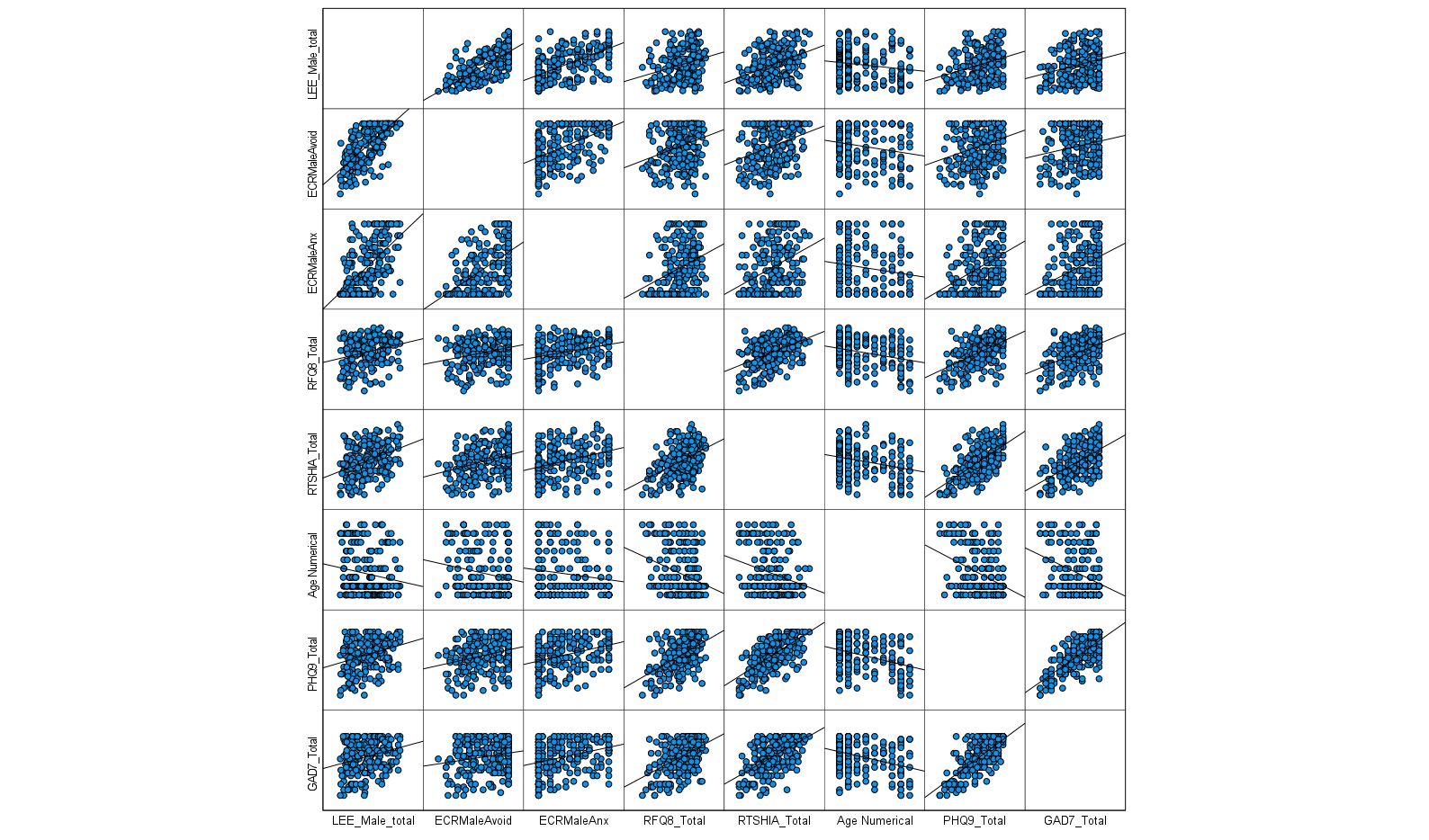


^N.B. Categorical covariate of gender not included in the scatterplot matrix.^

**Normality of residuals.** The mediation analyses presented in the main body of this article employed bootstrapping methodology. All estimates of direct and indirect effects reported in the paper are bootstrapped estimates, which follow asymptotic normally distributed parameter estimation drawn from bootstrapped resampling, thus providing an additional safeguard for the assumption of normality of residuals. Taken together with the below histograms and P-P plots, it can be inferred that the assumption of normality of residuals was met for both the male and female dataset.

Figure 6. *Histogram of regression standardized residuals for the dependent variable of RTSHIA_total in the female caregiver model*
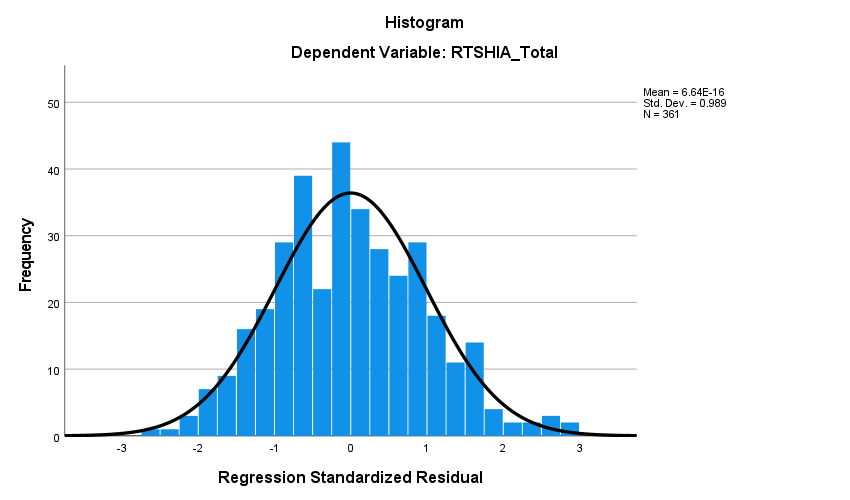


Figure 7. *Normal P-P plot of regression standardized residuals for the dependant variable of RTSHIA_total in the female caregiver model*


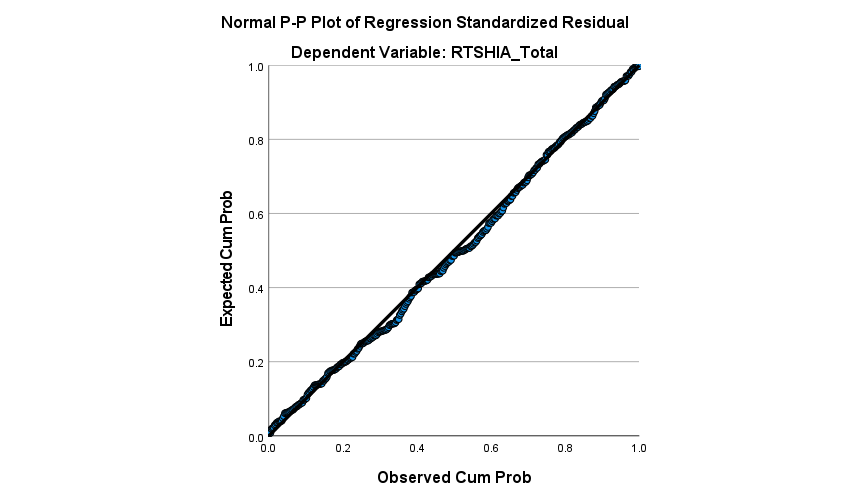


Figure 8. *Histogram of regression standardized residuals for the dependent variable of RTSHIA_total in the male caregiver model*


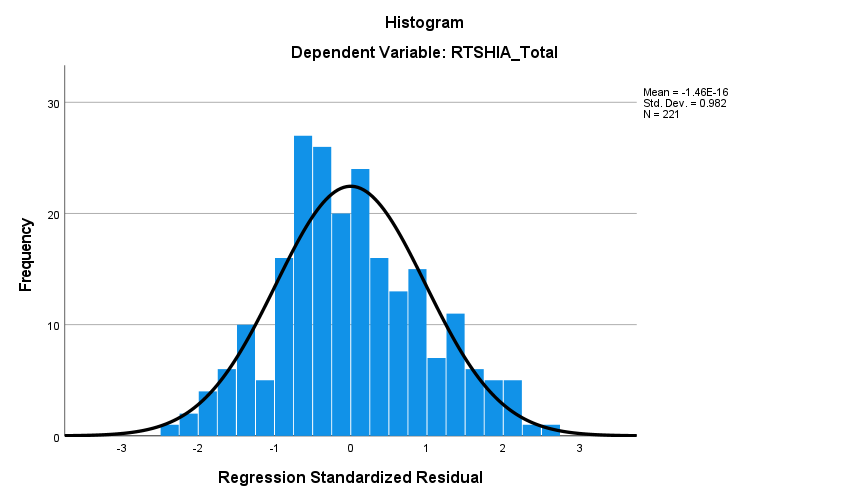


Figure 9. *Normal P-P plot of regression standardized residuals for the dependant variable of RTSHIA_total in the male caregiver model*


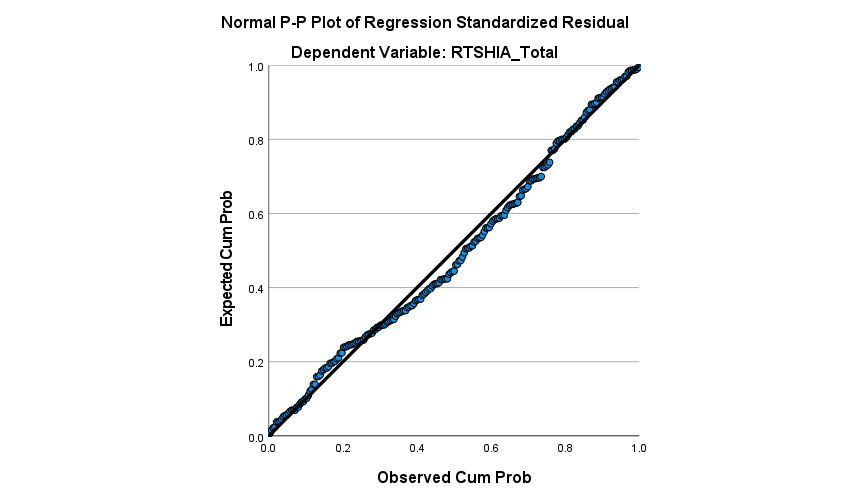


**Homoscedasticity.** As indicated by the below Figures, when plotted against each other, the standardized residuals and standardized predicted values all fell between -3.00 and 3.00 in both the male and the female dataset, with visualizations of the data indicating that the assumption of homoscedasticity had been satisfied.

Figure 10. *Scatterplot plotting the regression standardized residuals against the regression standardized predicted values for the dependent variable RTSHIA_total for the female caregiver dataset*


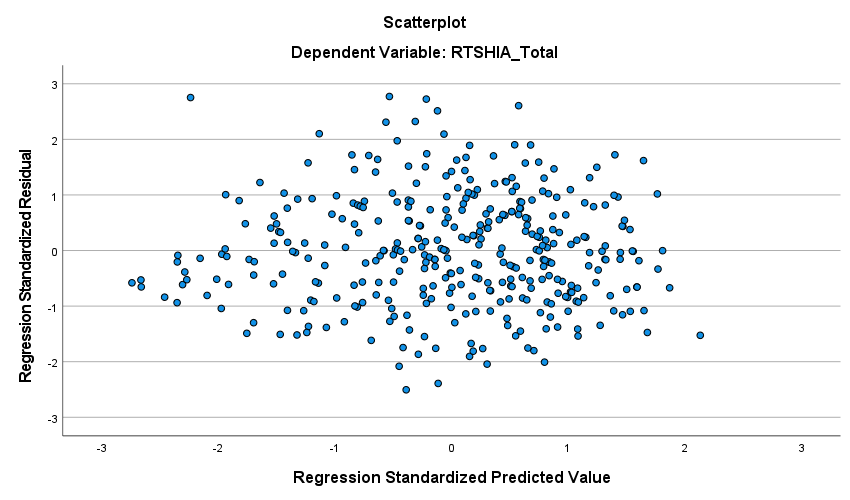


Figure 11. *Scatterplot plotting the regression standardized residuals against the regression standardized predicted values for the dependent variable RTSHIA_total for the male caregiver dataset*


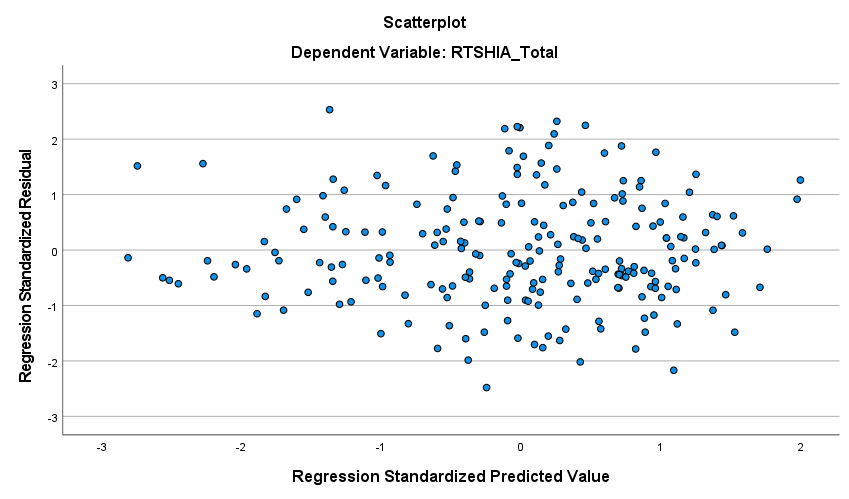


**Independence of errors.** Taken together with the visualisation of the data displayed above in Figure 10, the data in the female caregiver model meets the assumption of independence of errors (Durbin-Watson value = 2.138). Taken together with the visualisation of the data displayed above in Figure 11, the data in the male caregiver model meets the assumption of independence of errors (Durbin-Watson value = 2.055).
